# Metabolic profiling to evaluate the impact of amantadine and rimantadine on the secondary metabolism of a model organism

**DOI:** 10.1038/s41598-023-43540-w

**Published:** 2023-10-05

**Authors:** Marianna Kostina-Bednarz, Joanna Płonka, Hanna Barchanska

**Affiliations:** Department of Inorganic Chemistry, Analytical Chemistry and Electrochemistry, Faculty of Chemistry, Silesian University of Technology, B. Krzywoustego 6, 44-100 Gliwice, Poland

**Keywords:** Health care, Chemistry

## Abstract

Metabolic profiling offers huge potential to highlight markers and mechanisms in support of toxicology and pathology investigations during drug development. The main objective was to modify therapy with adamantane derivatives: amantadine and rimantadine, to increase their bioavailability and evaluate the influence of such therapy on drug metabolism using *Saccharomyces cerevisiae* as the model organism. In this study, the profile of endogenous metabolites of a model organism was measured and interpreted to provide an opportunity to investigate changes induced by treatment with amantadine and rimantadine. It was found that resveratrol supplementation synergistically enhanced the effects of amantadine treatment and increased rimantadine metabolism, potentially reducing side effects. The fingerprinting strategy was used as an efficient technique for qualitatively evaluating and monitoring changes in the profiles of endogenous components and their contents in a model organism. Chemometric tools were employed to find marker compounds that can be defined as characteristic indicators of a pharmacological response to a therapeutic intervention. An improved understanding of the mechanisms involved in drug effect and an increased ability to predict individual variations in the drug response of organisms will improve the treatment process and the development of new therapies.

## Introduction

Amantadine (AMT, adamantan-1-amine) and rimantadine (RMT, 1-(1-adamantyl)ethanamine) are small, synthetic, tricyclic amines of the adamantane class. The structures and physicochemical parameters of AMT and RMT are presented in Table 1.

**Table 1:** Physico-chemical characteristics of AMT, RMT and RSV.

Compound	Systematic name	Structure	logK_ow_	Solubility in water, g/L	pK_a_
Resveratrol (RSV)	5-[(E)-2-(4-Hydroxyphenyl)ethen-1-yl]benzene-1,3-diol		3.10	0.003	8.99
Amanatadine (AMT)	Adamantan-1-amine		2.44	6.3	10.58
Rimantadine (RMT)	1-(1-adamantyl)ethanamine		3.34	0.3	10.43

PubChem, accessed March 2023.

Based on literature studies, therapeutic applications of both AMT and RMT have evolved over the years. Initially, these compounds were employed as antiviral drugs. In the 1960s, it was discovered that AMT works by blocking M2 ion channels, inhibiting viral entry into cells and inhibiting viral replication^1^, whereas RMT interferes with the uncoating of viral RNA of the influenza-A virus, therefore both were recommended for the treatment of this type of influenza^2^. However, both of them are no longer recommended for the treatment of influenza-A infections, especially the H3N2 strain, as these have shown resistance to adamantanes^3,4^. AMT has been an established treatment for Parkinson’s disease for more than 50 years, both as monotherapy and as an adjunct to levodopa. AMT and RMT are low-affinity, non-competitive antagonists of NMDA receptors, moreover, they exhibit anti-dyskinetic properties by acting on several neurotransmitter systems, including the dopaminergic and glutamatergic systems^5^. However, due to acute side effects (inter alia, visual hallucinations, confusion, blurred vision, edema of the legs, dry mouth, superficial punctate keratitis, corneal endothelial dysfunction, and corneal edema), neither AMT nor RMT are preferred agents for Parkinson’s disease and are recommended only for patients that are less responsive to front-line treatments^5,6^. AMT may be used to support the treatment of depressive disorders and catatonia^1,7^. Due to the COVID-19 coronavirus pandemic, there have been a tremendous number of reports on the possibility of using AMT for the treatment of this disease. Conclusively, AMT is not recommended for the treatment of COVID-19^8^ therapy. The main problems with the effective and safe administration of AMT and RMT in the treatment of the aforementioned disorders appear to be their low bioavailability and, despite intensive research, a lack of a clear explanation of the drug’s mechanism of action^9–14^.

Along with the increasing efforts of societies to achieve sustainable development, there has long been an increased interest in plant-derived substances as potential (pro)drugs^15^. Resveratrol (RSV, 3,4′,5-trihydroxy-trans-stilbene) is a natural antitoxin widely distributed in grapes, berries, peanuts, green ferns, and other plants^16^. The RSV structure and physicochemical parameters are presented in Table 1.

RSV acts on different biological targets and thus produces different biological effects, therefore, it is being considered as a potential drug for the treatment of vascular diseases^17^, neurodegenerative diseases^18^, inflammatory disorders^19^, atherosclerosis, and cancer^20,21^. According to the latest reports, RSV has a beneficial influence on the coronavirus disease (COVID-19)^22^. The main limitation of the application of RSV as a drug is its incompletely understood mode of action, poor solubility in water, and low bioavailability after oral administration, while rapid after intravenous administration^23^. These factors are likely responsible for the fact that the clinical benefits of RSV have not been unequivocally proven to date^23^.

Current studies are being conducted to clarify the problems in the widespread application of RSV, AMT, and RMT in animals (zebrafish, rats, mice)^17,18,24^ and humans^6,25^. These organisms are complex, and thus the results obtained and the conclusions drawn from them may be the combined effect of many other factors in addition to the drugs applied. This problem is particularly relevant when studies are conducted on individuals suffering from other diseases than those potentially requiring AMT, RMT, or RSV therapy. Therefore, drug metabolism studies are also conducted on cell lines^20,21^ or using simple model organisms. An extensive discussion of the use of yeast as a model organism for higher eukaryotes, including humans, was conducted by van der Klei and Veenhuis^26^. They concluded that, despite some doubts, *Saccharomyces cerevisiae* is a suitable model organism for higher organisms^26,27^. To date, *Saccharomyces cerevisiae* as a model organism was employed in the study of various respiratory dysfunctions^28^, aging processes^29^, and neurodegenerative^30^ and metabolic^31^ disorders.

The available literature usually focuses on a certain process (changes in genes, metabolism of selected endogenous compounds) that occurs in the organism during a disease and treatment^32–34^. However, it should be borne in mind that both the disease and the treatment administered may affect the entire body, resulting in side effects. Therefore, based on *Saccharomyces cerevisiae* as a model organism, the purpose of our study was to (i) attempt to modify AMT and RMT therapy to increase their bioavailability, (ii) evaluate, using metabolic profiles and fingerprint analyses, the influences of the applied therapy on the secondary metabolism of the model organism, and (iii) establish if the application of RSV, as a pro-drug with potential therapeutic properties similar to AMT and RSV, could support the therapeutic process.

The conclusions of this study will contribute to the understanding of the mechanism of action of AMT and RMT and, thus, to their effective use in the treatment of neurodegenerative and metabolic diseases, as well as other disorders. The novel approach to holistically assessing treatment-induced changes in the body’s metabolism will be an important contribution to research on the causes of side effects associated with various therapies.

## Materials and methods

### Chemicals and reagents

AMT and RMT standards were supplied by Sigma Aldrich, Germany. Standard stock solutions with concentrations of 1.0 mg/mL were prepared in methanol. Analytical standards of L-phenylalanine (L-PHE), 5-hydroxy-L-tryptophan (5-HTRF), tryptamine (TRYP), L-tryptophan (L-TRF), dopamine (DA), DL-normetanephrine (NMN), DL-norepinephrine (NE), epinephrine (E), 5-hydroxytryptamine (5-HT), 5-hydroxyindole-3-acetic acid (5-HIAA), tyramine (TRA), L-tyrosine (L-TYR), 3,4-dihydroxy-l-phenylalanine (L-DOPA), *p*-coumaric acid (pCA), *trans*-cinnamic acid (tCA), and resveratrol (RSV) (purity > 99%) were also purchased from Sigma Aldrich, Germany. Individual stock solutions with a concentration of 10.0 mg/mL were prepared in an aqueous solution of 0.05 M HCl with the addition of 5 g/L Na_2_S_2_O_5_. All standard solutions were stored in amber glass vials at 4 °C in the dark. Working standard solutions were prepared daily by diluting the stock solutions in acetonitrile.

Acetonitrile (ACN), methanol (MeOH), formic acid (FA), acetic acid (AA), and water (LC–MS grade) were obtained from VWR, Germany. Analytical grade Na_2_S_2_O_5_ and HCl (conc.) were obtained from STANLAB, Poland. Nylon syringe filters (0.45 μm, 25 mm, PURELAND, CHEMLAND, Poland) were used to filter the sample extracts. Pharmaceutical-grade glucose powder was supplied by Hasco-Lek S.A., Poland, and the yeast *Saccharomyces cerevisiae* strains were obtained from Lallemand, Poland.

### Sample preparation

#### Incubation conditions

**Figure 1:**
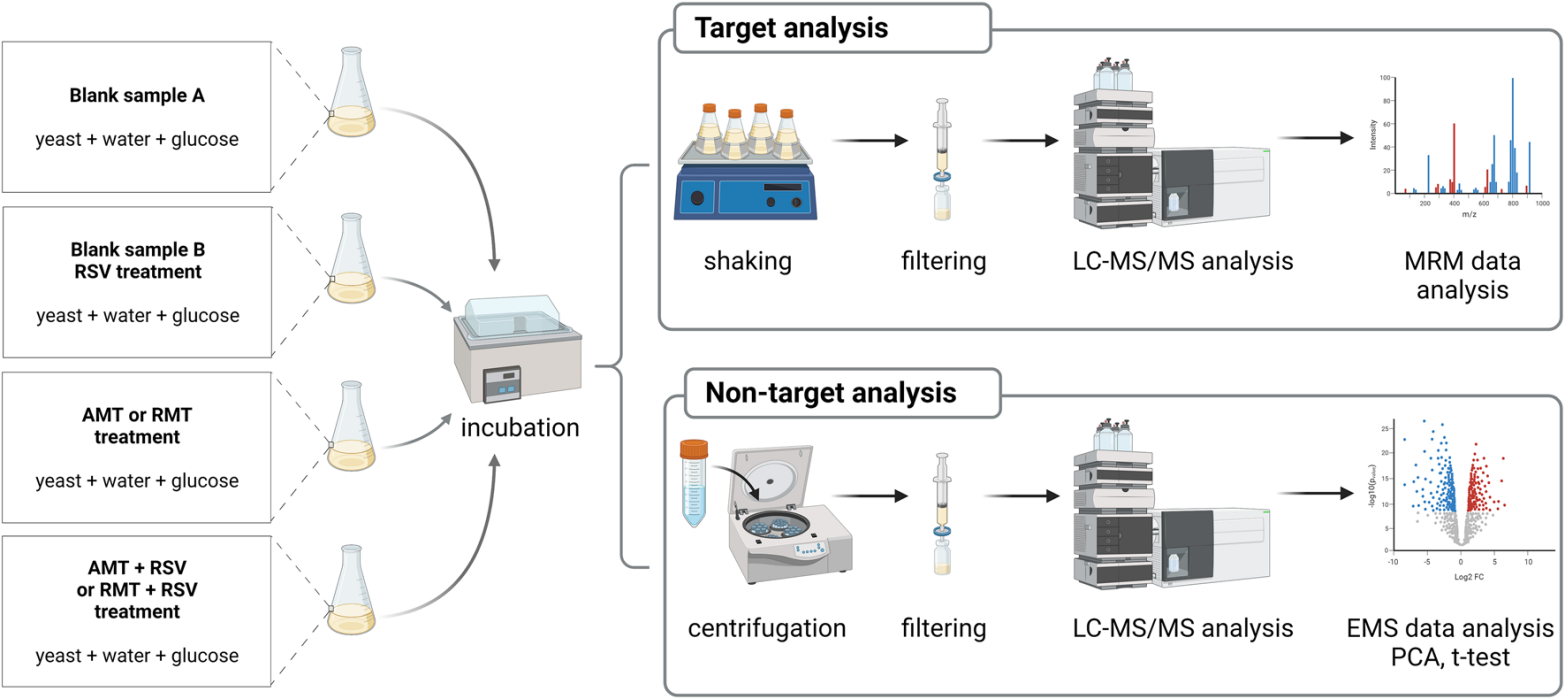
Schematic of sample preparations for target and non-target analysis (created with https://www.biorender.com/).

Water (LC–MS grade) was added to the yeast at a proportion of 1 mL H_2_O/5 g yeast and then homogenized. Every half-hour of the experiment, a glucose solution was added to the yeast samples at 12.0 µg/gram of yeast to support metabolic processes. The yeast was exposed to AMT, RMT, and RSV separately at a concentration of 4 µg/gram of yeast, equivalent to a therapeutic dose of AMT. A series of samples were exposed to AMT with RSV and RMT with RSV at the same doses. The treated material was incubated at 36 °C—a temperature similar to the human body. Yeast samples not exposed to any compounds were carried out under the same conditions and constituted the blank sample. Each incubation experiment was performed in three independent replicates. A schematic of sample preparations for target and non-target analysis is presented in Fig. 1.

#### Sample preparations for the target analysis

Target analysis was conducted to determine, in yeast, the selected endogenous compounds involved in the L-TYR, L-TRF, and L-PHE metabolic pathways, as well as exogenous compounds—AMT and RMT. The weighted material in 5 ± 0.01 g portions was mixed with 5 mL of 8 mM FA in ACN and then shaken for 1 h at 500 rpm. Subsequently, each sample was filtered through a nylon filter with a pore diameter of 0.22 μm, and 1 mL of the clear extract was transferred to a vial for chromatographic analysis. Detailed information on the optimization of extraction conditions is available in our article^31^. The procedure was repeated for all sample types in triplicate.

#### Sample preparations for the non-target analysis

Non-target analysis was performed to establish the fingerprint of yeast incubated under different conditions. The sample (1 ± 0.01 g) was mixed with 5.0 mL of methanol and then centrifuged for 20 min, 4000 rpm, at 20 °C. After centrifugation, the samples were filtered through a nylon filter (0.22 μm), and the obtained extract was diluted to 1:100, 1:1000, and 1:10,000 (v/v) with 8 mM FA in water immediately before analysis.

### LC–MS/MS instrumentation and conditions

The chromatographic separations were performed on a Dionex UHPLC system (Dionex Corporation, Sunnyvale, CA, USA), equipped with an UltiMate 3000 RS (Rapid Separation) pump, an UltiMate 3000 autosampler, an UltiMate 3000 column compartment with a thermostable column area, and an UltiMate 3000 variable wavelength detector. All of these components were operated using Dionex Chromeleon™ 6.8 software. The analytes were separated using a TSKgel ODS-100 V column (150 × 4.6 mm, 5 μm particle size, TOSOH Bioscience, Tokyo, Japan). The mobile phase consisted of FA solution in acetonitrile (8 mM, eluent A) and aqueous FA solution (8 mM, eluent B). The final analytical conditions for the LC elution gradient and flow rate are described in Table 1SM in the Supplementary Materials.

Mass spectrometric analyses were performed using an AB SCIEX 4000 QTRAP triple quadrupole mass spectrometer (Applied Biosystems/MDS SCIEX, Foster City, CA, USA) equipped with an electrospray ionization (ESI) source. The mass spectrometer was equipped with a Turbo Ion Spray™ ion source and was used in positive and negative ion modes. The source-dependent operating parameters were optimized to obtain the best performance from the mass spectrometer for the analysis of compounds. These parameters for target and non-target analyses were the nebulizer gas and curtain gas pressures, the collision gas, the ion spray voltage, and the temperature of the heater gas, for details, see Table 2SM.

AMT, RMT, and selected compounds from the L-TYR, L-TRF, and L-PHE metabolic pathways were determined in yeast extracts by LC–ESI–MS/MS using the Multiple Reaction Monitoring (MRM) mode. Because of the high selectivity of detection, MRM was used for target analyte quantitation, where the analyte was identified and determined specifically through the combination of the parent mass and unique fragments. The highest and second-highest abundance transitions were used for quantification and confirmation, respectively. The use of electrospray ionization for profiling L-TYR, L-TRF, and L-PHE has enabled the formation of intact molecular ions, which greatly assists the screening of unknown and target analytes in complex sample matrices. The tuning and optimization of the compound-dependent parameters, including the declustering potential (DP), collision energy (CE), entrance potential (EP), and collision exit potential (CXP), were performed through the direct infusion of a standard solution into the ion source using a Harvard syringe pump. The pairs of monitoring ions for AMT and RMT, as well as compound-dependent MS parameters, are shown in Table 3SM, while selected endogenous compounds were presented in our previous article^31^. The protonated molecular ions [M^+^H]^+^ of AMT and RMT at the optimized conditions of QTRAP MS were at m/z 152.108 and 180.644, respectively, for details, see Supplementary Material, Figure 1SM. The method has been validated, and the validation parameters are described in the Supplementary Materials, Table 4SM.

Non-target analysis was conducted using the enhanced ion trap scanning mode mass spectrometry (EMS) with positive and negative ionization. In EMS, the ion trap is filled with molecular ions generated from the ion source, and the ions are spread out axially to the detector, to identify unknown compounds. Ions are transmitted from the source through the quadrupoles into the ion trap. This provides a full scan analysis of all the analytes entering that QTRAP system. In the non-target strategy, chromatographic conditions were chosen to elute the entire spectrum of compounds characterized by different polarities. To achieve this, a relatively long column was used that allowed the analysis to run for 20 min to separate a large number of compounds from the test sample. A gentle gradient mobile phase system, starting with a solvent of low elution strength and gradually changing to a solvent of higher strength, was also used to aid chromatography detection. A quality control (QC) sample was prepared to validate the measurement stability and robustness. The QC sample was analyzed after each set of nine real samples in both positive and negative metabolomic data acquisition modes.

### Post-acquisition data analysis

The software used to operate the mass spectrometer was Analyst (Version 1.5.1, Applied Biosystems, Foster City, CA). The programs MarkerView (v. 1.1, Sciex, US) and MetaboAnalyst 5.0. was used for post-acquisition data processing. Statistical significance was determined using a Student’s *t*-test and ANOVA (*p* < 0.05).

### Evaluation of the correctness of the statistical methods

**Figure 2:**
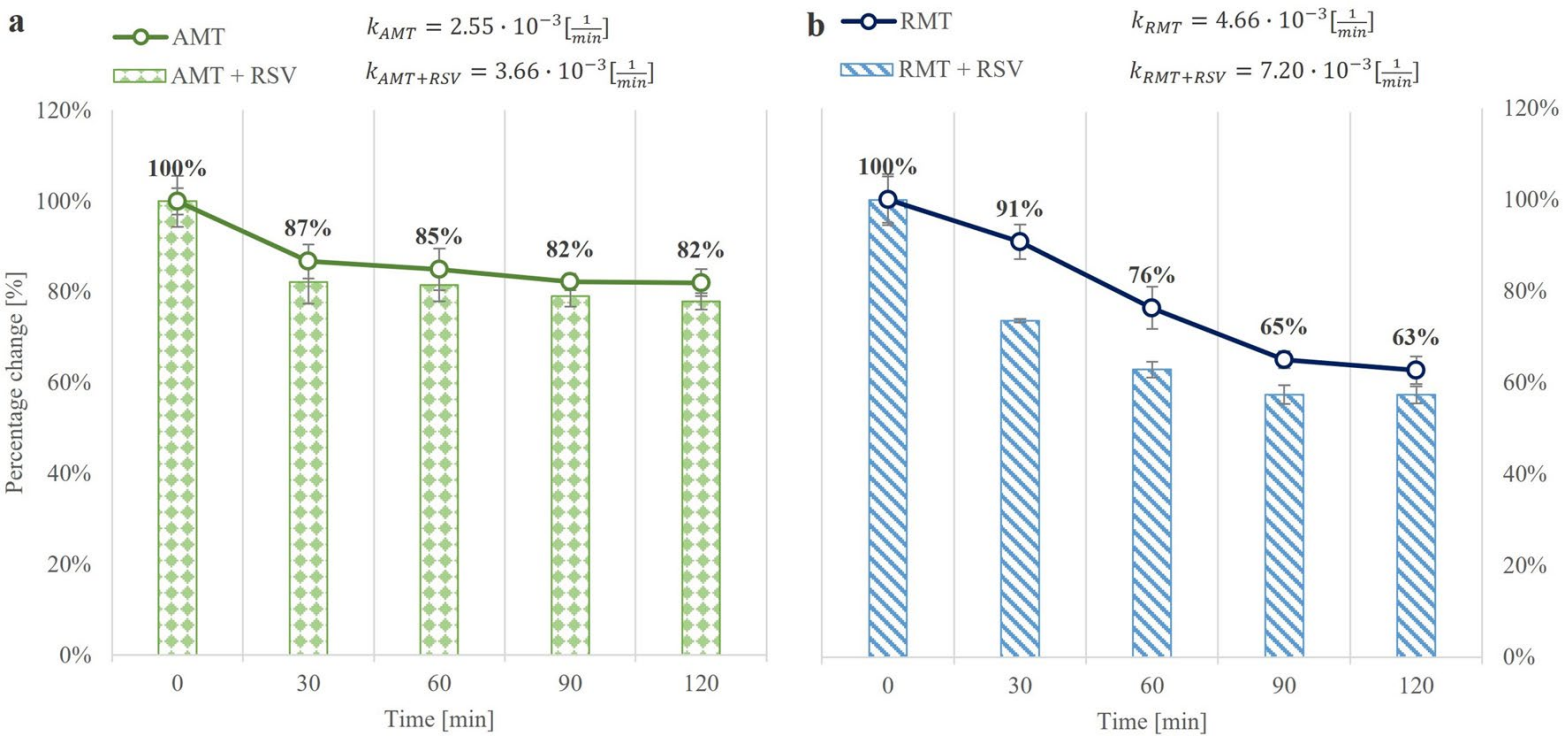
Dissipation curves of (**a**) AMT and (**b**) RMT. The bar graphs show the changes in the concentration of the adamantine derivatives after the addition of RSV. Data are expressed as mean (n = 3); error bars represent CVs.

Yeast metabolic profiles were obtained based on target analysis conducted in the ion trap MRM mode with positive and negative ionization. A dataset of selected endogenous compounds (Table 5SM) was assessed using the web-based software, MetaboAnalyst 5.0, to identify affected metabolic pathways that could be altered by the use of AMT and RMT (*p* < 0.05). The graphs (Figure 2SM) of metabolic pathways of (a) *Saccharomyces cerevisiae* and (b) *Homo sapiens* were made by plotting on the y-axis the − log10 transforms of *p*-values from the pathway enrichment analysis and on the x-axis the pathway impact values derived from the pathway topology analysis. The color of each pathway is determined by the *p* value (red = lowest *p* value and highest statistical significance), and the node radius (size) is based on the pathway impact factor, with the biggest indicating the highest impact. According to this analysis, the four most altered pathways were distinguished, which are common to the species *Saccharomyces cerevisiae* and *Homo sapiens*. These pathways are tyrosine metabolism, tryptophan metabolism, phenylalanine metabolism, and the biosynthesis of phenylalanine, tyrosine, and tryptophan. A detailed pathway analysis table, including the identified pathways, *p* value, and false discovery rate is presented in Supplementary Table 6SM. Because of the high similarity of metabolic pathways found in these two species, it can be concluded that yeast is a plausible example of a model organism for research to understand basic metabolic processes in humans.

MarkerView Software 1.2.1 (AB SCIEX) was used for data processing and chemometric analysis (data mining, alignment, and principal component analysis–discriminant analysis [PCA-DA]). This software was used for the visualization of many types of data with principal component analysis (PCA). This tool is an unsupervised multivariate statistical analysis approach that allows trends to be recognized across groups of samples within a dataset and does not consider the experimental groups when interpreting the raw data, only the overall variation. PCA-DA—a supervised version of PCA—can also be used, where previous knowledge of sample groups is employed to determine the variables that maximize the variation between groups and those that minimize the variation within a group. PCA results are commonly plotted in two- or three-dimensional plots that reflect the behavior of the samples (scores plot) or variables (loadings plot). The scores plot highlights the largest variation in the dataset as the first principal component (PC1, x-axis) and the second largest variation as PC2 (y-axis). The principal components are calculated in order of the amount of variance they explain, PC1 and PC2 should account for most of the variance in the data, and it is possible to use the Scores plot to detect clusters, outliers, and trends in the data. Whereas the loading plot analysis provides insight into the variables that lead to sample trends in the performance graph and illustrates which relationships are being adjusted upwards or downwards.

Data mining was performed with an automated algorithm in the retention time (RT) range of 0.5–18 min, the parameters for peak detection were as follows: noise threshold, 5000 counts; minimum chromatographic peak width, 3 scans; minimum spectra width, 0.2 Da; background subtraction offset, 10 scans; and subtraction multiplication factor, 1.3. An RT tolerance of 0.3 min and a mass tolerance of 0.4 Da were established for peak alignment and filtering. Two separate positive and negative ionization data matrices, comprising lists of peaks characterized for each sample were obtained according to RT, m/z, intensity, and charge state. The data set was analyzed using PCA-DA. Before the actual PCA, most of the data were pre-processed using Pareto scaling. This scaling technique subtracts the mean from each variable and divides it by the square root of the standard deviation. Pareto scaling shows a particularly good suit for mass spectrum data processing. MarkerView Software offers the flexibility to use different scaling algorithms, such as mean centering, and autoscaling, providing more flexibility in data analysis, which have also been used.

Another statistical analysis tool applied to the research data was the “*t*-test”. The *t*-test is a supervised analysis technique and is useful when two or more predetermined classes of samples are present. The pair-wise *t*-test finds features that are discriminatory between 2 groups of samples. It is useful when datasets are complex with many samples and sample types, and the *t*-test can narrow down specific features that are more relevant between individual groups. Plotting the treated/control fold change of each compound versus the *p* value, the compounds with the most significant differences can be easily visualized. A pair-wise comparison of all compounds was conducted, or the relation of one compound to all the others was compared. The results of the *t*-test indicate how well each variable distinguished the two groups. This is reported as a *p* value, i.e., the probability that the observed difference occurs by chance. The most appropriate way to visualize these results is to plot the *p* values computed for each variable versus its log fold change. This is known as a volcano plot and helps illustrate how large and how significant the specific variable is in distinguishing between the two groups. For any two groups, the volcano plot shows the log of the fold change (the ratio of the average response in each group) as a function of the *p* value (the probability that the observed difference occurs by chance) for each variable. Especially significant variables are those that have a low *p* value (small probability of occurring by chance) and a large fold change. At the extremes of the horizontal axis are the groups of variables that are completely absent in one sample group, that is, they have an infinite fold change. The changes for significant variables can be easily visualized, with response plots for the variables across the different samples and box and whisker plots.

**Figure 3:**
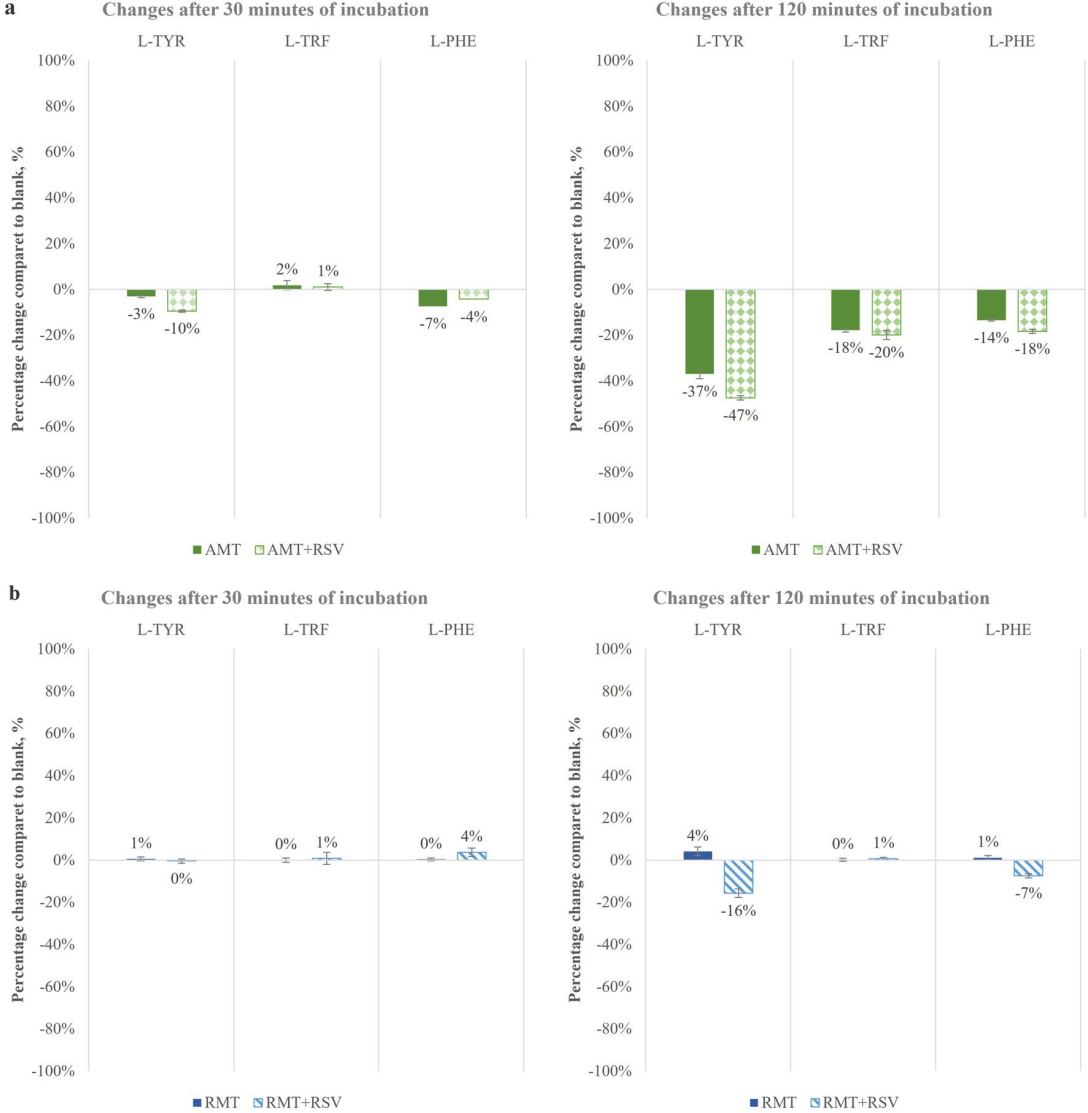
Percentage changes in the concentrations of L-TYR, L-TRF, and L-PHE in yeast exposed to (**a**) AMT and (**b**) RMT after 30 and 120 min of incubation. Blank was taken as 0%. Data are expressed as mean (n = 6); error bars represent CVs.

The exported data from target and non-target analysis were also preprocessed by MetaboAnalyst 5.0. The data was normalized, Pareto-scaled, and log-transformed before analysis to reduce the impact of large feature values and make all features more comparable or normally distributed. Benjamini–Hochberg correction was applied throughout to account for multiple test comparisons. One-way ANOVA tests were performed on MetaboAnalyst for all data with post-hoc analysis by both Fisher’s Least Significant Difference method (Fisher’s LSD) and Tukey’s Honestly Significant Difference (Tukey’s HSD), utilizing an FDR value cutoff of 0.05 to note significant compounds. Tables 7SM, 8SM, and 9SM included in Supplementary Materials give numerical details from the ANOVA. Sortable lists of *p* values and the −log10 of the *p* values are given along with the False Discovery Rate (FDR)–adjusted *p* values (based on the Benjamini–Hochberg procedure). Figures 3SM, 4SM and 5SM shows the important features identified by ANOVA analysis.

## Results and discussion

### Preliminary studies

The following preliminary studies were performed to establish the conditions for researching the influence of AMT and RMT on yeast metabolism. To select the glucose dose and the time of incubation, two sets of yeast treated with AMT (4 µg/g) were prepared. Both of these sets were spiked every 30 min with glucose, however, one set was spiked at 1.2 µg/g, while the second was at 120 µg/g. Every 30 min, yeast samples were taken and AMT was determined in them. The experiment was carried out until the yeast activity stopped. In this manner, the yeast was found to be active, regardless of the glucose dose, during two hours of incubation. In the next step, the influence of the glucose dose on AMT metabolism was established. For this purpose, two sets of yeast treated with AMT (4 µg/g) were prepared, and glucose was added to one set at a dose of 1.2 µg/g and to the other set at a dose of 120 µg/g. The incubation was carried out for two hours and samples in which AMT was determined were taken every 30 min.

Degradation rate constants were calculated as values proportional to the decrease in the initial concentration of the drug as a function of time. The kinetics of the AMT degradation reaction following the addition of glucose solution at two different concentrations were calculated, and the data was plotted as a percentage of the remaining drug as a function of time, an example of the AMT dissipation curve is presented in Fig. 2. The reaction rate value was calculated according to first-order mechanics. During the study, no strong degradation of AMT was observed over time, regardless of the concentration of glucose added. The reaction rate during the addition of glucose at a concentration of 1.2 µg/g yeast was 0.0011 1/minute, while at a concentration of 120 µg/g yeast, it was 0.0012 1/minute. In accordance with the above studies, the yeast was incubated at 1.2 µg/g for up to 120 min in subsequent tests. As a result of the lack of yeast activity, no significant differences in AMT metabolism were observed. Studies on RMT metabolism were conducted in the same way.

### Effect of RSV on AMT and RMT metabolism in a model organism

The interaction between AMT and RSV and RMT and RSV were investigated in a further experiment. The research was performed according to the procedures described in “Incubation conditions” and “Sample preparations for the target analysis” Sections. Yeast samples were exposed to AMT or RMT and in parallel to a mixture of AMT and RSV or RMT and RSV in a dose of 4 μg of each compound/gram of yeast. Samples for chromatographic analysis were taken every 30 min for two hours of yeast incubation to determine the influence of compounds overtime on the metabolism of the model organism. Metabolism of AMT administered separately occurs slowly (${k}_{AMT}=2.55\cdot  {10}^{-3}\frac{1}{min}$) and a significant decrease in the concentration of this drug occurred 30 min after exposure and then remained constant. In a parallel experiment in which RSV was added to AMT, no significant changes in AMT metabolism were observed ${(k}_{AMT+RSV}=3.66\cdot  {10}^{-3}\frac{1}{min})$, which is illustrated in Fig. 2a.

Compared to the dissipation of AMT, the degradation rate of RMT is higher ${(k}_{RMT}=4.66\cdot  {10}^{-3}\frac{1}{min}$). As shown in Fig. 2b, the metabolism of RMT by yeast is faster if the drug is administered in a mixture with RSV compared to the exposure of a model organism to RMT alone ${(k}_{RMT+RSV}=7.20\cdot  {10}^{-3}\frac{1}{min}$), which may be indicative of higher metabolism. The addition of RSV to RMT has a statistically significant effect on increasing the rate of metabolism of this drug in the first 60 min after dosing, but the disappearance curve stabilizes from that point on. Presumably, the reason for the faster metabolism of RMT is its increased bioavailability and biotransformation, which prevents it from accumulating in cells and thus causing side effects. Therefore, it can be hypothesized that the risk of toxic effects due to the excessive solubility of these substances is reduced by the addition of RSV. RSV enhances RMT metabolism, so it can be assumed that RSV supplementation during RMT therapy will be beneficial.

### Yeast metabolic profiles

Following the procedures described in “Incubation conditions” and “Sample preparations for the target analysis” Sections, a series of matrix extracts were prepared to determine the overall metabolic responses of yeast exposed to selected exogenous compounds. The model organism was treated individually to AMT, RMT, a combination of AMT with RSV, or RMT with RSV in three repetitions. A non-spiked extract of yeast served as the blank sample A (BLK A) and was used to compare changes in the composition of endogenous compounds induced by the exposure of yeast to AMT, RTM, and RSV, in various combinations, with the concentrations of these natural compounds in an unexposed sample. Simultaneously, a reference sample was prepared for comparison purposes, marked as blank B (BLK B), which was exposed only to RSV. According to the conclusions obtained in the study of drug dissipation curves (Fig. 2), samples for chromatographic analysis were taken after 30 and 120 min of incubation to determine the effect of the compounds over time on the metabolism of this organism. Determination of selected endogenous compounds involved in the metabolism of L-TYR, L-TRF, and L-PHE was performed by quantitative analysis by comparing changes in the content of these compounds in exposed samples to blank samples. Of all endogenous compounds included in this study, there were statistically significant changes (*t*-test at the significance level of 5%) in the L-TYR, L-TRF, and L-PHE concentrations in yeast exposed to AMT or RMT. The changes in the concentrations of these amino acids were expressed as percent changes in yeast samples exposed to AMT or RMT compared to the blank A samples and AMT + RSV or RMT + RSV compared to the concentrations of these compounds in blank B samples, as presented in Fig. 3. After 30 min of incubation, no significant changes caused by the exogenous compounds were observed. A slight reduction (at the level of 10%) in the content of L-TYR was induced by AMT administered in the mixture with RSV. Nevertheless, in samples taken after 120 min of incubation, significant AMT-induced changes were observed in the endogenous compound contents of L-TYR (-37%), L-TRF (− 18%), and L-PHE (− 14%), and were enhanced after the addition of RSV to − 47%, − 20%, and − 18%, respectively. It can be assumed that, due to the slow release of AMT and its long presence in the model system, AMT induces more changes in the metabolic pathways of endogenous compounds compared to the effects of RMT, as shown in the data presented in Fig. 3. RMT induce no substantial changes, and the addition of RSV to RMT resulted in only a 16% reduction in L-TYR content after 120 min of incubation. This slight effect of RMT on the organism may be a result of its faster metabolism and shorter exposure time.

**Figure 4:**
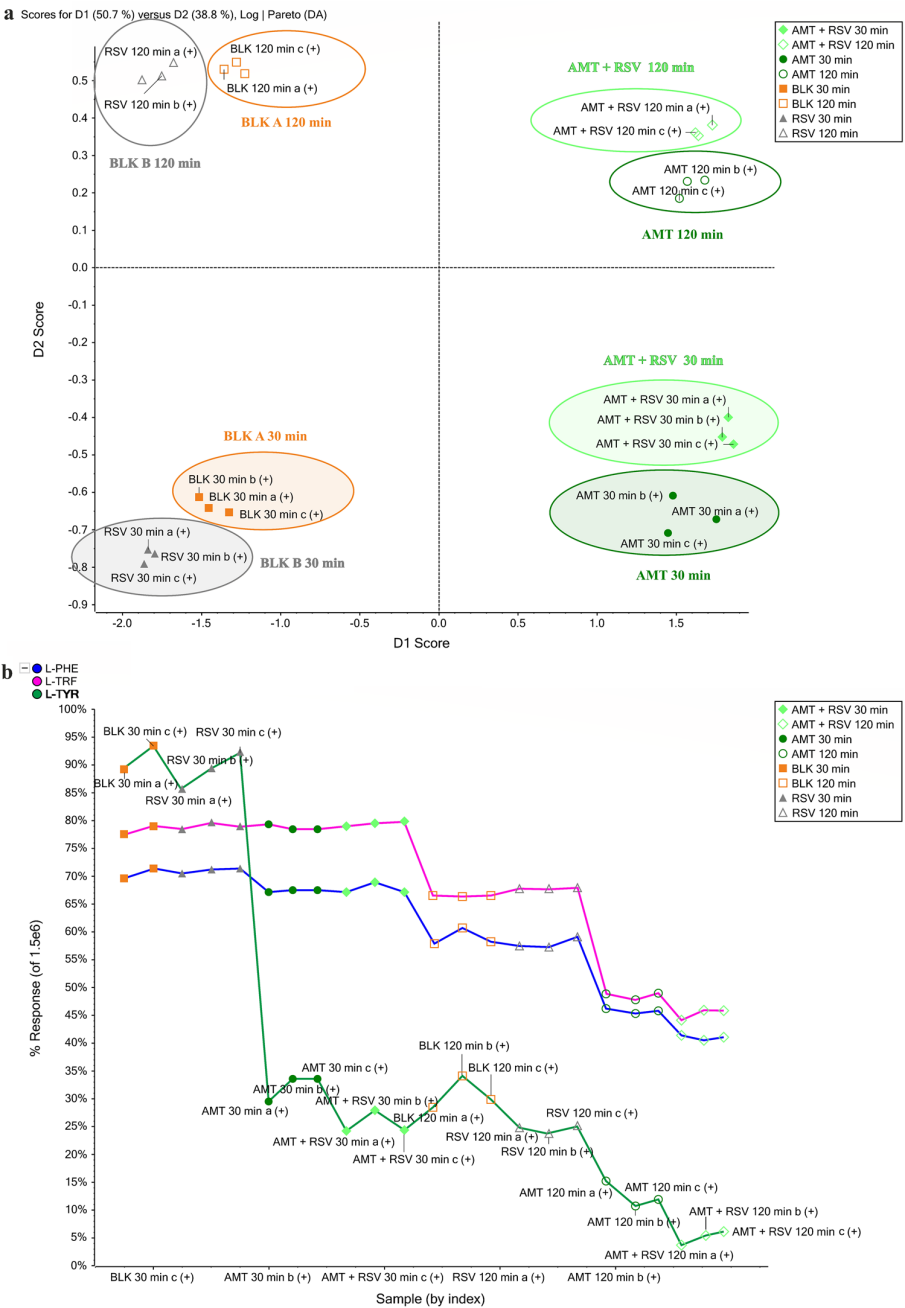

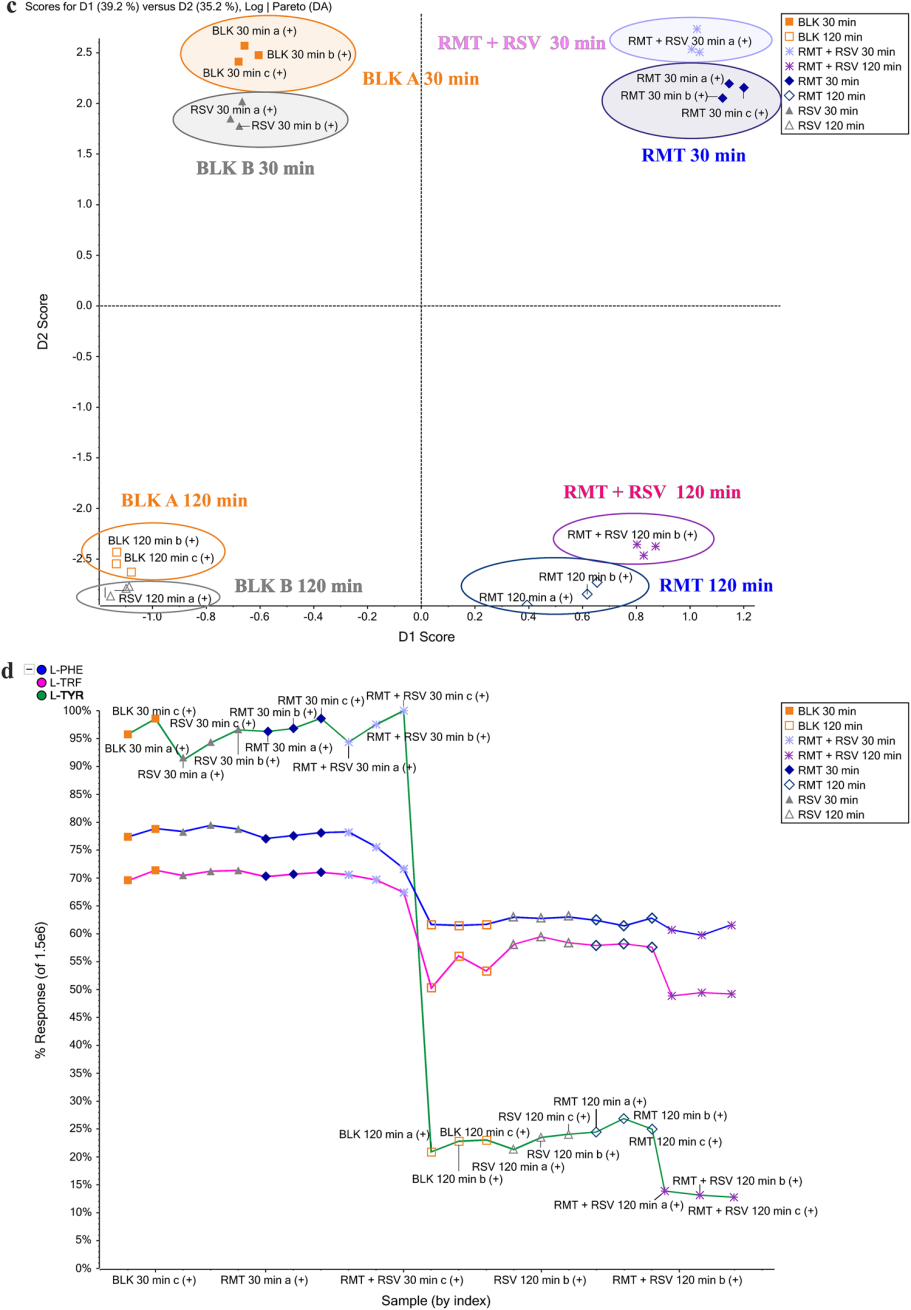
The scores plot (**a**) and profile plot (**b**) for AMT and the scores plot (**c**) and profile plot (**d**) for RMT generated from MRM data in positive ionization mode.

PCA data visualization tools have been applied to gain valuable insight into any trends in yeast metabolism induced by AMT and RMT treatment. The procedure for selecting the parameters of the statistical methods is described in “Post-acquisition data analysis” section. Metabolic profiling was performed using MRM data from the target analysis, samples from the three replicates were sorted by time of sampling (30 min or 120 min incubation) and by exposed compound (AMT, RMT, AMT + RSV, or RMT + RSV). These results were compared with blank A (unexposed) and blank B (exposed only to RSV) samples. Chemometric tools were used to identify which features are unique to the different types of samples (for example, exposed to AMT), investigate differences between sample sets (for example, exposed to AMT and RMT), and characterize the constituent profile of complex unknowns. All peaks from the MRM MS data from positive ionization modes have been shown in the PLS-DA loadings plot and provide a rationale for the sample separations observed in the scores plot. The scores plot (Figs. 4a,c) shows sample grouping and the responses of compounds with similar trends, while the loadings plot reflects the variables causing the responses to separate. Analyzing the MRM MS data obtained from the target analysis, it was observed that the four groups were differentiated by principle component analysis grouping. The yeast metabolic response as a result of AMT treatment (green dots) appears tightly clustered and cleanly separated in the plot from the blank samples (orange squares) and the metabolic response to RSV treatment (gray triangles). The loadings plot was used to quickly find features that differentiate samples and shows the feature identifier, such as m/z plus RT. The found features are distinctly present in the blank sample group and are therefore identified as unique for this organism.

The loadings plot was selected to generate and display the profile plot shown in Fig. 4b, which shows the response of the samples. Profile plots were generated to show the intensity of selected compounds across multiple samples to assist in finding changes in yeast metabolism. In the case of this model organism, these compounds were identified as L-TYR, L-TRF, and L-PHE. As seen from the scores plot, blank samples A formed a compact cluster and are in the graph’s corner, as are the responses to RSV (blank B), and are close to them after both 30 and 120-min incubations. The metabolic responses of the organism in blank A and B samples differentiate due to incubation time, which shows the metabolic processes of the organism occurring during the study. After exposure to AMT alone and AMT with RSV, the metabolic responses of the organism are comparable. The profile plot shows a strong decrease in L-TYR content as a result of AMT exposure after 120 min of incubation, which is even more intense after RSV addition. This trend also occurs in the changes in L-TRF and L-PHE content, but the reducing effect is weaker than that of L-TYR. This information is confirmed by quantitative analysis, the results of which are shown in Fig. 3. In the curve (Fig. 4b), the points for AMT at 120 min and AMT + RSV at 120 min are below BLK at 120 min and RSV at 120 min, showing that the content of endogenous compounds was reduced. A strong reduction occurred in the content of L-TYR, therefore, in the profile graph, the response lines after AMT and RSV exposure are the lowest among the rest of the points. Analyzing the same graph in the next step observed that the response lines (the AMT at 120 min and AMT + RSV at 120 min points) on the response graph are almost at the same level, and the addition of RSV increased the reducing effect. The results provide excellent confirmation of the interaction between AMT and RSV, which can be described as synergistic since they increase their influence on yeast metabolism when administered in a mixture. In the PCA plot, the metabolic response of the organism after exposure to AMT and RSV shows a strong separation from the responses induced by the individual effects of each compound and the nonexposed sample.

An analogous experiment was carried out in which the metabolic response of the organism treated with RMT alone and combined with RSV was studied. In this example, PCA again simplified the visualization of complex data sets for exploratory analysis, and the results were evaluated by examining score plots that showed the variable responses for samples treated with AMT and RMT in a mixture with RSV, as well as blank samples (Fig. 4c). Statistical data processing proved that it was possible to identify the effect of the treatment of yeast with RMT or RMT + RSV after 30 and 120 min of incubation within the blank samples. The profile plot (Fig. 4d) shows the response for the samples as the intensity of the selected endogenous compounds. This graph showed no change in response as a result of the effect of RMT on yeast metabolism after both 30 and 120 min of incubation, while the addition of RSV to RMT after 120 min resulted in a slight reduction in L-TYR of 18% and L-PHE of 7% according to the data obtained from the quantitative analysis. The curves on the profile graph of changes in endogenous compounds represent a significant change in the metabolic responses of the organism under incubation.

### Yeast metabolic fingerprints

**Figure 5:**
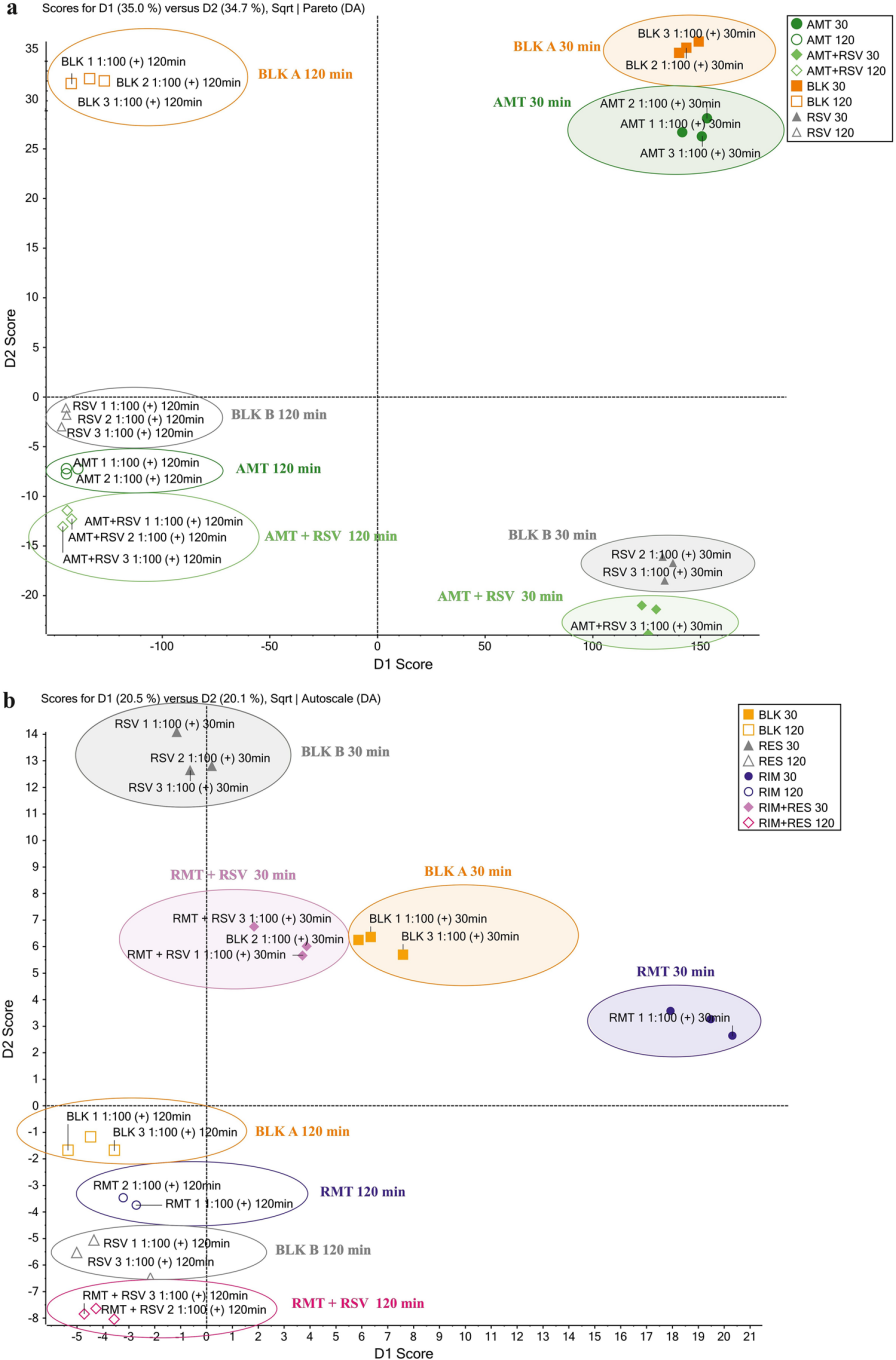
Scores plot showing the metabolic response of the organism after exposure to AMT (**a**) and RMT (**b**) regarding the blank A and blank B samples.

Based on non-target analysis data, an explorative effort assessed the overall response of organisms after AMT and RMT, and the combination of these compound treatments with RSV, allowing any possible clustering in a supervised modality. The research was performed according to the procedure described in “Incubation conditions” and “Sample preparations for the non-target analysis” Sections. The metabolic responses induced by these exogenous compounds were compared to those obtained in blank A (unexposed) and blank B (RSV-exposed) samples. The full scan data were processed to find confirmation for authenticity exposition for AMT, RMT, or RSV. The results are shown in Fig. 5 for the positive ionization mode. The clusters of metabolic responses to each compound are indicated on the score plot as ellipses. If the ellipses on the PCA plot do not overlap, these groups were considered separate clusters. The fingerprinting strategy provided an unambiguous identification of treatment with adamantane derivatives in the model organism because of the marked metabolic response of the organism after exposure to these compounds. The separate clusters were visualized, indicating that PCA-DA satisfactorily segregates the response after the addition of RSV from the rest of the samples after 30 and 120 min of incubation. In both Fig. 5a,b, the metabolic fingerprint of the organism obtained after 30 min of incubation was strongly separated from that obtained after 120 min, which is additional evidence of changes in the organism’s metabolism during this process.

As presented in Fig. 5a, after AMT treatment and 30 min of incubation, the model organism induces a similar metabolic response (green dots) as that obtained for blank sample A (orange squares). This confirms the target analysis in which no significant changes were observed in the contents of endogenous compounds after AMT exposure at the beginning of the incubation period. However, after 120 min of this process, the situation changed, and AMT induced significant changes in the contents of L-TYR, L-TRF, and L-PHE. The marked separation of the metabolic response of the unexposed organism from the samples exposed for 120 min of incubation demonstrates the changes induced by these compounds. These effects are strongest as a result of yeast treatment with AMT + RSV, as evidenced by the greatest remoteness of the metabolic fingerprint elicited by this mixture from the unexposed sample.

Using a metabolic fingerprinting strategy, it was also possible to identify the metabolic response following RMT treatment by unambiguously separating the metabolic response induced by this compound from those obtained in blank and AMT-exposed samples (Fig. 5b). Regarding the blank samples, there were no significant changes after RMT exposure, as shown by the close alignment of the metabolic response in the unexposed sample (orange squares) to the exposed RMT (blue dots). This trend is preserved after both 30 and 120 min of incubation. When comparing the metabolic responses of the model organism induced by AMT and RMT, compounds with similar structures and actions, their different effects were observed. After 120 min of incubation, the metabolic response of AMT treatment was very distant from the response of the untreated sample, while the responses induced by RMT were closely located in the blank sample. This is confirmed by the quantified percentage changes in L-TRF, L-PHE, and L-TYR concerning the blank presented in Fig. 3. AMT exposure increased the content of these compounds after 120 min of incubation, and the addition of RSV enhanced this effect, particularly for L-TYR concentrations.

**Figure 6:**
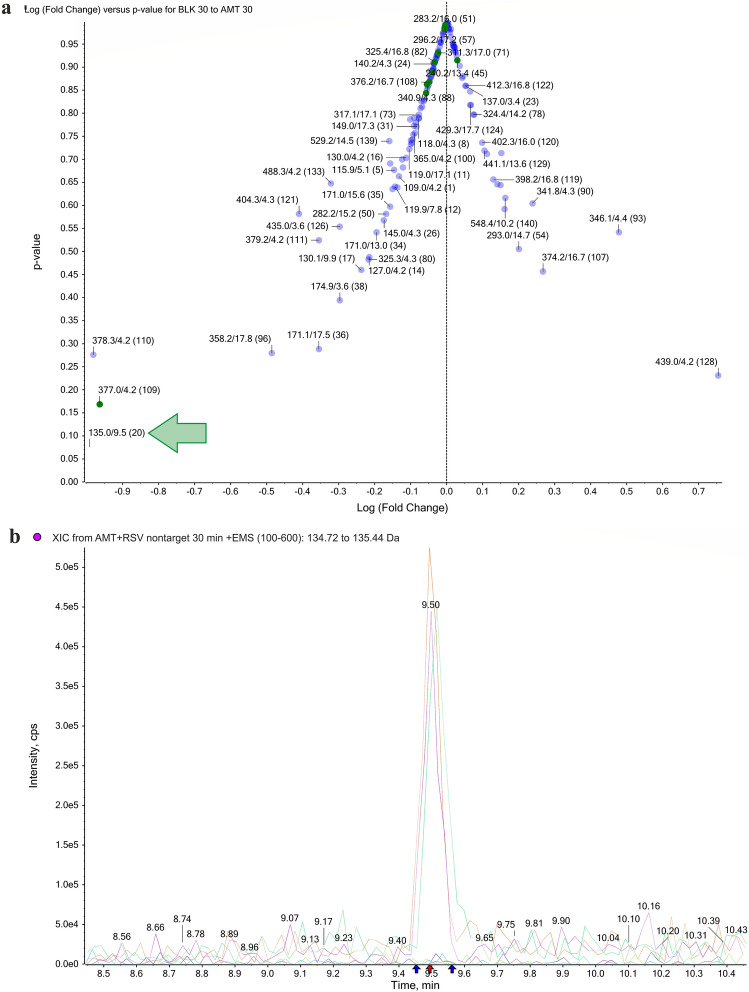

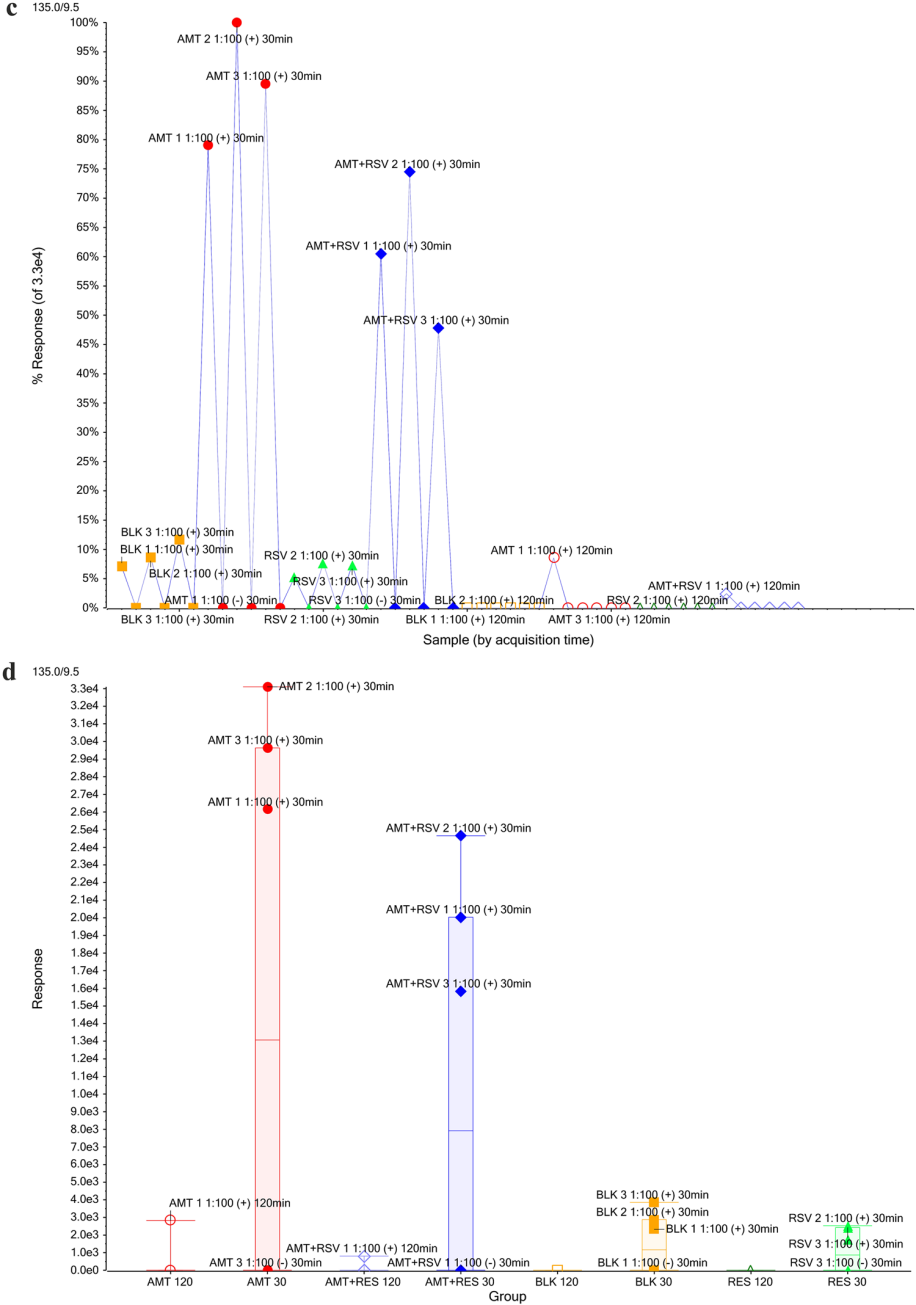
T-test analysis comparing features between different samples after exposure to AMT. The volcano plot (**a**) highlights features with the greatest fold change and statistical significance. Chromatogram of MRM transition for AMT (**b**). The computer whisker plot **(c**) and the response profile (**d**).

To find the components that made the greatest difference to the chemical composition between blank samples and those after treatment, the samples in the clusters were divided into the corresponding groups, after which a comparison of the groups was performed using a *t*-test. As a result of this comparison, the variables (i.e., m/z of molecular ions) were obtained, which were ranked in order of increasing values of p, which indicates the probability of the presence of the corresponding substance (m/z) simultaneously in both groups. After ranking the obtained list of substances, their distribution by samples was studied. In Fig. 6a, the panel shows all the data, such that features with a low p-value and high fold change differ in abundance between the groups with high confidence. One peak, shown by the arrow in the volcano plot (Fig. 6a), strongly differentiating the AMT response from others is circled and added to an interest list for identification. Manual extraction of the ion current performed in the Analyst software (XIC) showed that a molecular ion with a mass of 135.0 was found in samples taken after the exposition of AMT. The chromatogram confirmed a peak from the ion (135.0) at a RT of 9.5 min (Fig. 6b). After selecting the most extreme feature from the volcano plot (m/z 135.0 at 9.5 min), it generated a profile plot for all samples (Fig. 6c) and a box-and-whisker plot (Fig. 6d) for experimental groups. The peak with m/z 135.0 corresponded to the adamantane group formed by the degradation of AMT, and the ion was absent in blank samples but present in AMT-treated samples. This peak was much more abundant in the AMT treatment samples than in other samples.

**Figure 7:**
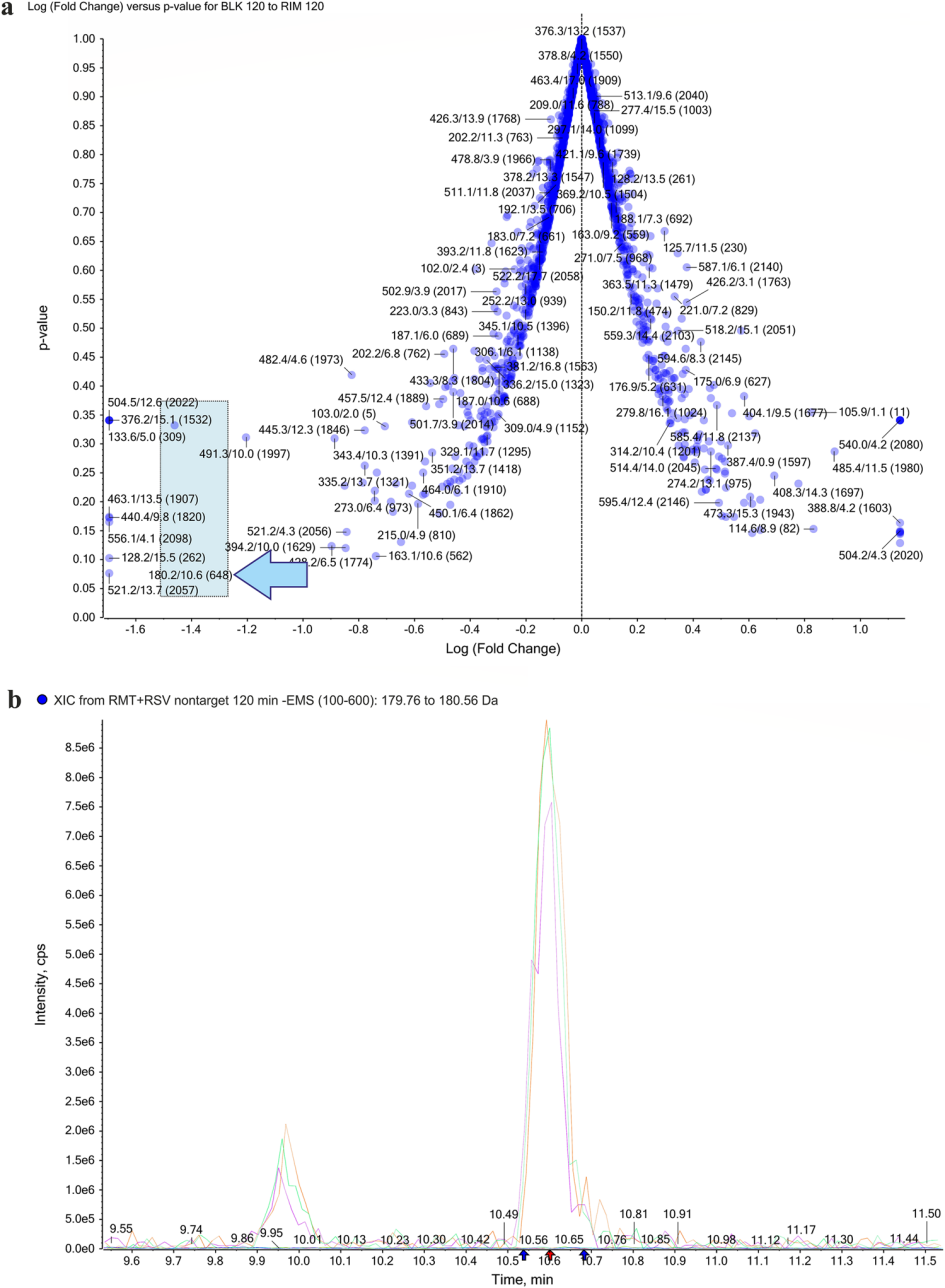

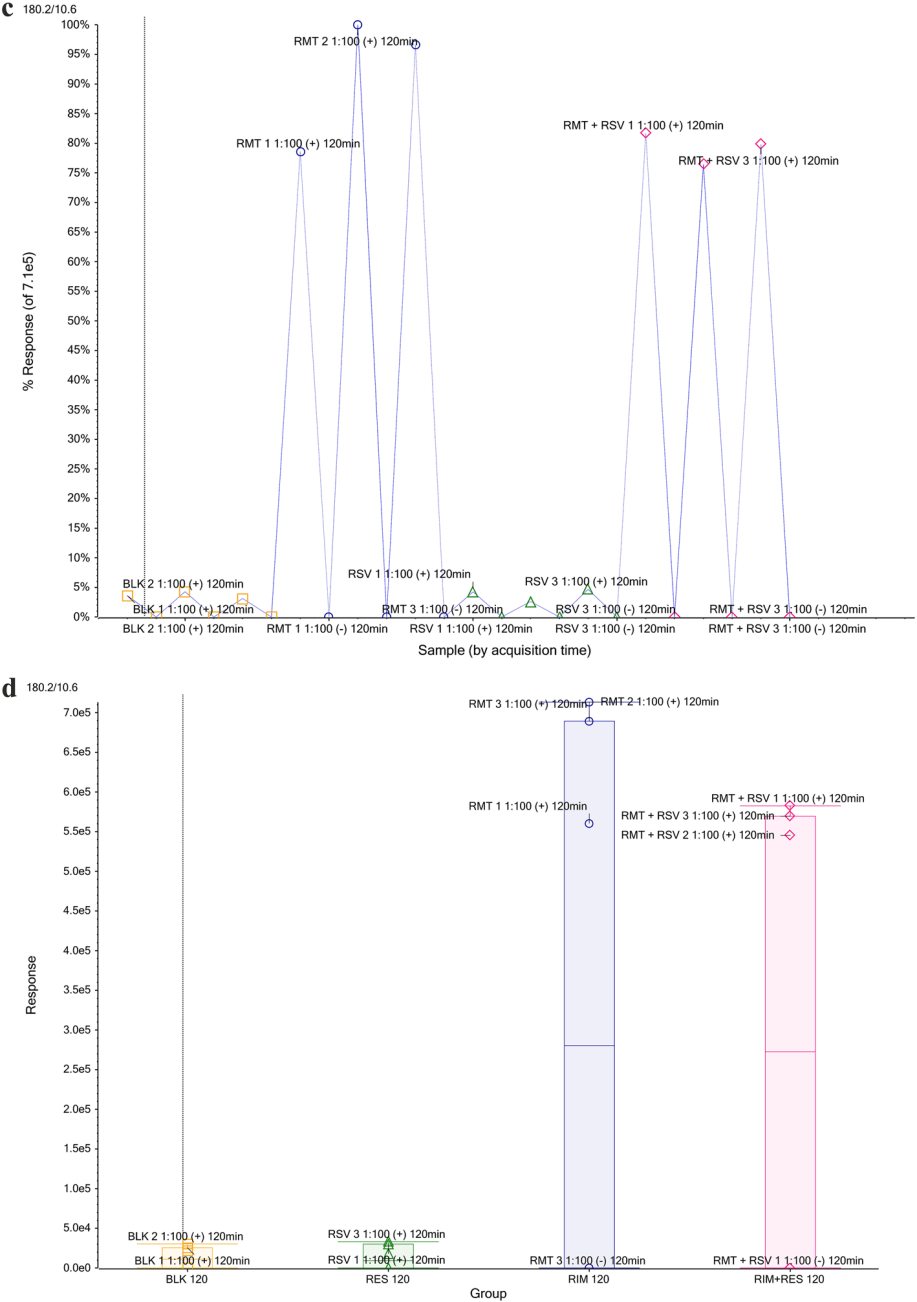
T-test analysis comparing features between different samples after exposure to RMT. The volcano plot (**a**) highlights features with the greatest fold change and statistical significance. Chromatogram for MRM transition for RMT (**b**). The computer whisker plot (**c**) and the response profile (**d**).

The same statistical analysis was carried out for data obtained after exposure of the organism to RMT and RMT with RSV. The volcano plot, illustrated in Fig. 7a, allowed us to observe both how large and how significant the specific variables are in distinguishing between the yeast’s metabolic responses to RMT treatment and blank samples. The lowest *p* value and a large fold change had a point of 180.2/10.6. This occurs at the end of the horizontal axis and corresponds to the responses after RMT exposure and corresponds to the RMT ion. A chromatogram with a selected peak from ion 180.2 at a RT of 10.6 min confirmed the presence of this characteristic ion in RMT-exposed samples (Fig. 7b). After selecting this characteristic ion, response profile plots were generated (Figs. 7c and 7d), which also proved the differentiation of the experimental samples studied due to RMT exposure. Therefore, it can be considered a characteristic marker of treatment with this drug. The significance of fingerprint results was statistically based on ANOVA analysis. The important features selected by ANOVA plot with *p* value threshold 0.05 for samples exposed to AMT and RMT are presented in Supplementary Figures 4SM and 5SM respectively. All features that occurred after AMT or RMT exposure were identified by one-way ANOVA, and the parameters of the 10 most important are presented in Supplemental Tables 8SM and 9SM. This additional analysis provided a more comprehensive perspective on the validity of results, and the outcomes aligned with the conclusions drawn from the *t*-test analysis.

## Conclusions

The polyphenol nutraceutical RSV, due to its antiviral properties and anti-Parkinsonian effects, fits well with those of AMT and RMT, thus making it a promising candidate as a pharmaceutical synergist. Structurally, there are three phenolic hydroxyl groups in an RSV molecule, which provides the ability to participate in non-covalent interactions with their hydrogen-donating and/or -accepting capabilities. It is especially important for chloride ions of pharmaceutical hydrochlorides, such as AMT or RMT, through charge-assisted hydrogen-bonding interactions between phenolic hydroxyl groups and chloride ions^35^. In research, to reduce the side effects of the conventional antiviral drug AMT or RMT hydrochloride by down-regulating its excessive dissolubility and gaining synergistically enhanced effects, the approach of bidirectional optimization has been discovered. Preliminary studies have investigated the effects of adding RSV on AMT and RMT metabolism. In this research, RMT’s advantage of high water solubility was used to enhance RSV’s dissolubility and bioavailability, thereby activating its auxiliary antiviral effects. Meanwhile, the excessive solubility of RMT was properly reduced. Furthermore, the risk of toxic effects caused by the excessive solubility of RMT is lower secondary to a slower release. In return, the pharmaceutical activity of RSV contributes cooperatively with RMT in treatment efficacy, achieving a synergistic effect. All these benefits open a new pathway for the development of synergistic pharmaceutical specifics. The natural antiviral bioactivity of RSV makes it a promising adjuvant with RMT, therefore, it was combined with this drug. Further exploration to evaluate the bidirectional optimization effects in this field will be interesting in future research.

In subsequent approaches, the concept of pharmacometabonomics was applied as “the prediction of the outcome of a drug intervention in an individual based on a mathematical model of pre-intervention metabolite signatures”^36^. The study illuminated the baseline and changes in treatment outcomes of three drugs with antiviral activity. The results reported in the article confirmed that AMT metabolism occurs more slowly than RMT, and no significant effect of RSV on this process was observed under test conditions. Therefore, AMT takes much longer to be eliminated in an exposed organism, so it can be assumed that it exhibits a stronger effect than RMT, which metabolizes quickly. This hypothesis is supported by target analysis, in which a quantitative analysis was performed to compare changes in the content of selected endogenous compounds involved in the metabolism of L-TRF, L-PHE, and L-TYR in exposed and blank samples. These studies proved that although RMT has a similar structure to AMT, it induces a different metabolic response in the model organism. The addition of RSV to both AMT and RMT resulted in a synergistic enhancement of the effect of each drug. This study provides important new insights into the influence of these compounds on the concentrations of L-TRF, L-PHE, and L-TYR, which play a key role in neurotransmitter biosynthesis. Our research showed that in yeast exposed to AMT after 120 min of incubation, there was a significant change in the content of selected endogenous compounds and the reducing effect was increased by the addition of RSV. RMT, due to its faster metabolism, therefore, shorter exposure time, did not cause any significant changes in the content of the tested endogenous compounds.

The performance of data analysis, as well as data processing for visualization and statistical evaluation, was used to assess the metabolic response of the organism after exposure to AMT and RMT. MS-based non-target analysis spectra were acquired in Enhanced MS mode in negative and positive polarity, and full scan data were processed using PCA to find markers for authenticity exposition for AMT and RMT. The discovered AMT (135.0/9.5) and RMT (180.2/10.6) markers may be defined as characteristic indicators of a pharmacological response to a therapeutic intervention. This research provided an approach to preclinical variability assessment, confirming that the methodology offers practical utility for rapid assessment of drug treatment. The results of this study will greatly contribute to further investigations on the effects of these drugs on the human body and the mechanism of action of AMT and RMT, which is especially important in the prevention of their undesirable side effects.

### Supplementary Information

Supplementary Information.

## Data Availability

The datasets used and/or analyzed during the current study are available from the corresponding author on reasonable request.
