# Supplementary material for: Metabolic profiling to evaluate the impact of amantadine and rimantadine on the secondary metabolism of a model organism
Source: Sci Rep. 2023 Oct 5;13:16822. doi: 10.1038/s41598-023-43540-w (PMC10555991; doi:10.1038/s41598-023-43540-w)
Supplement: Supplementary file 1 — Supplementary Information. [file 41598_2023_43540_MOESM1_ESM.pdf]

Supplementary Materials

Metabolic profiling to evaluate the impact of amantadine and rimantadine on the secondary metabolism of a model organism

Marianna Kostina-Bednarz [a,*], Joanna Płonka [a], Hanna Barchanska [a]

[a] Department of Inorganic Chemistry, Analytical Chemistry and Electrochemistry, Faculty of Chemistry, Silesian University of Technology, B. Krzywoustego 6, 44-100 Gliwice, Poland

* corresponding author: email: marianna.kostina-bednarz@polsl.pl

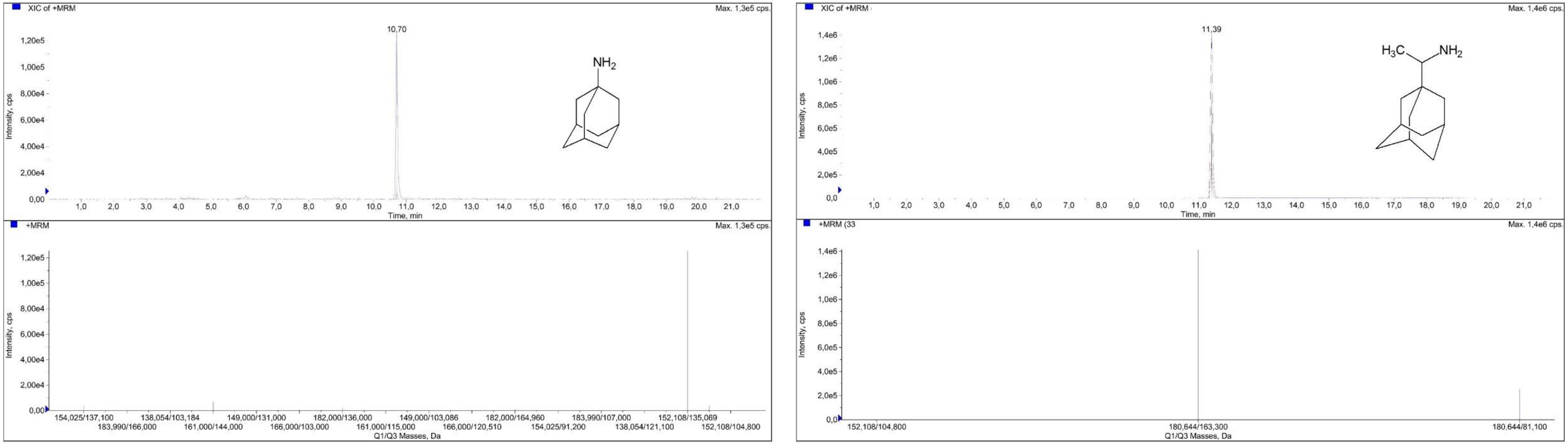

Figure 1SM Extraction of exact ion chromatograms and positive ion mass spectra of (a) AMT ([M+H]+, m/z 152.108) and (b) RMT ([M+H]+, m/z 180.644.

Table 1SM Time-scheduled gradient elution program

Type of analysis	Time [min]	Mobile phase composition		Flow rate [mL/min]	Temperature [°C]	Injection volume [μL]
		8 mM FA in ACN [%]	8 mM FA in H_2O [%]			
Target analysis	0.0	2	98	0.8	20	10
	4.5	2	98			
	15.0	100	0			
	15.1	2	98			
	20.0	2	98			
Non-target analysis	0.0	10	90	0.5	25	20
	15.0	100	0			
	15.1	10	90			
	20.0	10	90			

Table 2SM Source-dependent parameters for analysis

Type of analysis	Ion spray voltage [V]	Source temperature [°C]	Nebulizer gas [psi]	Heater gas [psi]	Curtain gas [psi]
Target analysis	4500	450	30	25	35
	-4500	450	30	25	35
Non-target analysis	4500	400	25	20	30
	-4500	400	25	20	30

Table 3SM The MS/MS parameters for AMT and RMT

Analyte	Q1 [a] [m/z]	Q3 [b] [m/z]	DP [c] [V]	EP [d] [V]	CE [e] [V]	CXP [f] [V]
AMT	152.1	135.1	76	9	25	10
		107.3	76		37	4
RMT	180.6	163.3	56	9	15	10
		81.1	56		29	6

a) Q1 – precursor ion. b) Q3 – fragment ion. c) DP – declustering potential.
d) EP – entrance potential. e) CE – collision energy. f) CXP – cell exit potential.

Validation

The selection of the assay conditions (extraction conditions, LC-MS parameters) of the developed methodologies was the result of a compromise between the high matrix complexity, its variations, the number of compounds to be determined in one analytical process in a short time, and the values of the validation parameters. Quantitative analysis determination of selected endogenous compounds involved in the metabolism of L-TYR, L-PHE, and L-TRF by comparing changes in the content of these compounds in exposed samples compared to blank samples. The methods developed for the target analysis of selected compounds in the model organism were validated and presented in our article[1], whereas the validation parameters of the method for the determination of AMT and RMT have been included in Table 4SM.

Linearity was assessed by assaying calibrations in blank yeast extracts at eight concentrations (the concentration range of 0.2–8.0 µg/mL). A calibration curve was constructed by plotting the ratio of the analyte peak area versus analyte concentration. The linearity of the calibration curve was evaluated by linear regression analysis. The limits of determination (LOD) and limits of quantification (LOQ) were evaluated from the concentrations of each compound in signal-to-noise ratios of 3 and 10, respectively. The accuracy of the method was presented as a recovery value with a standard deviation. Precision indicated the reproducibility, and it was determined as the percentage of the standard deviation from the arithmetic mean of the calculated sample concentrations and expressed as the percent of coefficient of variation (CV). Analyte recoveries were determined at three spiked compound concentration levels for the samples. The matrix effect (ME) was calculated as a ratio of the slope of the calibration curve of the analyte in the mobile phase and the blank sample extract (matrix-matched calibration plot). All the validation procedures were done with three replicates.

Table 4SM Method validation data

Analyte	Range [µg/mL]	[R^2]	LOD[a] [µg/mL]	LOQ[b] [µg/mL]	ME[c] [%]	Concentration [µg/mL]	CV[d] [%]	Recovery [%]
AMT	0.2 – 8.0	0.9960	0.0068	0.0204	82.0	1.0<br>4.0<br>8.0	3.2<br>4.3<br>2.3	87.8<br>82.1<br>78.5
RMT	0.2 – 8.0	0.9914	0.0025	0.0077	101.9	1.0<br>4.0<br>8.0	3.4<br>1.2<br>3.1	73.7<br>78.4<br>75.1
RSV	0.2 – 8.0	0.9931	0.0028	0.0084	75.9	1.0<br>4.0<br>8.0	2.0<br>2.1<br>0.9	85.5<br>88.3<br>86.3

a) LOD – limit of detection. b) LOQ – limit of quantification. c) ME – matrix effect. d) CV – coefficient of variation.

Table 5SM Summary of the identified endogenous compounds

No.	Compound	Chemical Formula	*m/z*	Adduct	Fragments (*m/z*)
11	Tyramine	$C_8H_{11}NO$	138.054	[M+H]+	103.184<br>121.100
15	*trans*-cinnamic acid	$C_9H_8O_2$	149.000	[M+H]+	131.000<br>103.086
6	Dopamine	$C_8H_{11}NO_2$	154.025	[M+H]+	137.100<br>91.200
5	Tryptamine	$C_{10}H_{12}N_2$	161.000	[M+H]+	144.000<br>115.000
14	*p*-coumaric acid	$C_9H_8O_3$	165.000	[M+H]+	146.900<br>118.900
1	L-Phenylalanine	$C_9H_{11}NO_2$	166.000	[M-H]+	120.510<br>103.000
8	DL-norepinephrine	$C_8H_{11}NO_3$	170.067	[M+H]+	152.000<br>107.100
4	5-hydroxy-L-tryptophan	$C_{11}H_{12}N_2O_3$	176.947	[M+H]+	160.200<br>115.100
3	L-Tyrosine	$C_9H_{11}NO_3$	182.000	[M+H]+	164.960<br>136.000
7	DL-normetanephrine	$C_9H_{13}NO_3$	183.990	[M+H]+	165.900<br>134.000
9	Epinephrine	$C_9H_{13}NO_3$	183.990	[M+H]+	166.000<br>107.000
13	5-hydroxyindole-3-acetic acid	$C_{10}H_9NO_3$	189.900	[M+H]-	145.900<br>143.900
12	L-DOPA	$C_9H_{11}NO_4$	197.800	[M+H]+	152.000<br>107.100
2	L-Tryptophan	$C_{11}H_{12}N_2O_2$	205.000	[M+H]+	188.000<br>146.000
10	5-hydroxytryptamine	$C_{10}H_{12}N_2O$	220.900	[M+H]+	204.100<br>162.000

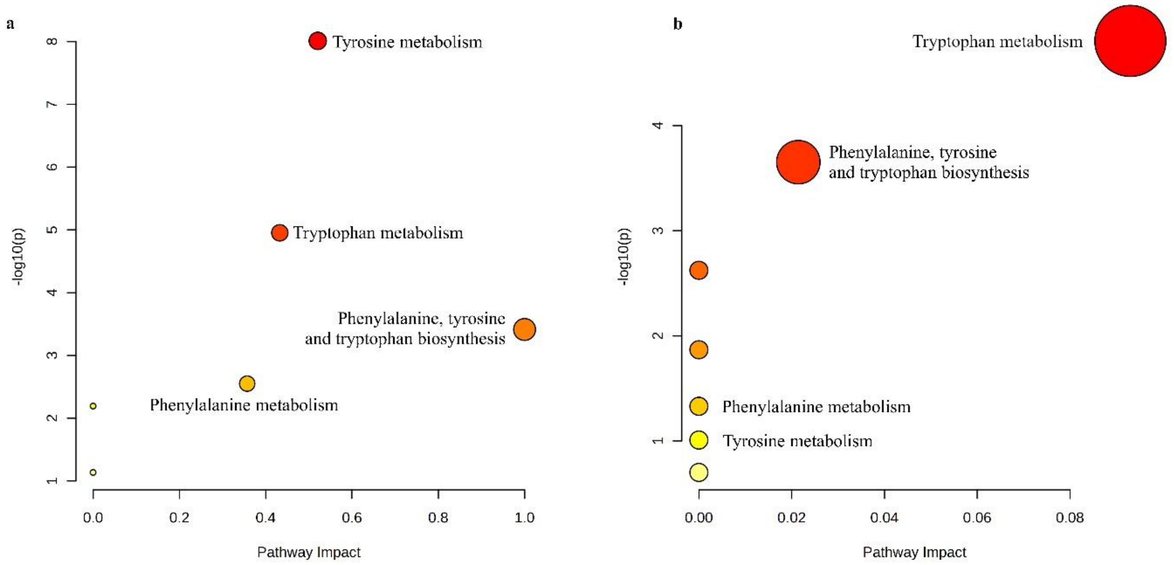

Figure 2SM Metabolic pathway analysis plot created for (a) *Saccharomyces cerevisiae* and (b) *Homo sapiens*.

Table 6SM Pathway analysis with significantly different metabolites using MetaboAnalyst

Pathway Name	*p*-value	-log(*p*)	FDR[a]	Impact
	Saccharomyces cerevisiae			
Tyrosine metabolism	0.098066	1.0085	1	0
Tryptophan metabolism	1.56E-05	4.8056	0.00114	0.09302
Phenylalanine, tyrosine and tryptophan biosynthesis	2.23E-04	3.6521	0.00813	0.02144
Phenylalanine metabolism	0.046814	1.3296	0.68349	0
	Homo sapiens			
Tryptophan metabolism	1.11E-05	4.9535	0.00047	0.43273
Phenylalanine, tyrosine and tryptophan biosynthesis	3.86E-04	3.4132	0.01081	1
Phenylalanine metabolism	0.0028149	2.5505	0.05911	0.35714
Tyrosine metabolism	9.7558E-09	8.0107	8.1948E-07	0.52078

a) FDR – *p*-value adjusted using False Discovery Rate

Table 7SM Important features identified by One-way ANOVA
for data obtained based on target analysis of yeast after exposure to AMT and RMT

Peaks (mz/rt)	*f*-value	*p*-value	-log10(*p*)	FDR
RMT 180.6/163.3_11.46	45.460	2.39E-13	12.621	4.07E-12
AMT 152.1/135.1_10.67	33.591	6.97E-12	11.157	5.93E-11
RSV 226.8/184.9_13.1	21.242	9.73E-10	9.0121	5.51E-09
TRYP 161.0/144.0_10.64	14.362	5.28E-08	7.2775	2.24E-07
pCA 165.0/146.9_10.35	12.699	1.75E-07	6.7561	5.96E-07
DA 154.0/137.1_4.57	10.993	6.90E-07	6.1613	1.95E-06
TRA 138.1/103.2_10.84	10.531	1.03E-06	5.9879	2.50E-06
L-PHE 166.0/103.0_9.22	9.2602	3.31E-06	5.4802	7.03E-06
L-DOPA 197.8/152.0_2.73	8.2431	9.21E-06	5.0359	1.74E-05
NE 170.1/152.0_1.76	7.2539	2.72E-05	4.5657	4.62E-05
NMN_184.0/165.9_2.92	7.0808	3.32E-05	4.4790	5.13E-05
E 184.0/166.0_2.92	6.7061	5.17E-05	4.2867	7.32E-05
L-TRF_205.0/188.0_10.26	4.6408	8.16E-04	3.0881	0.001068
L-TYR_182.0/136.0_7.16	4.5737	9.02E-04	3.0447	0.001096
tCA 149.0/131.0_13.66	3.1255	0.009468	2.0237	0.010730
5HIAA 189.9/145.9_10.6	3.0170	0.011464	1.9407	0.012181
5-HT 176.9/160.2_9.3	2.6682	0.021511	1.6673	0.021511

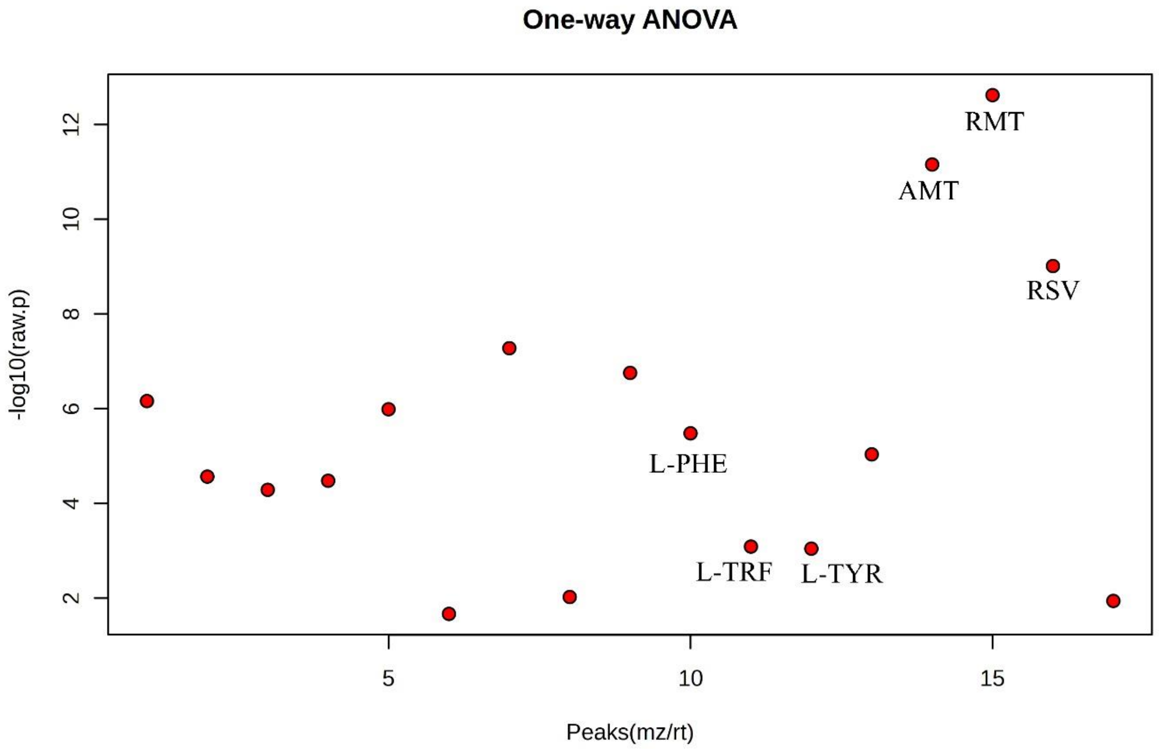

Figure 3SM Scatterplots of *p*-values obtained using the one-way analysis ANOVA for target analysis based on the default parameters. The points highlighted in red are the significant compounds selected based on the default *p*-value threshold (0.05). The figure was generated using MetaboAnalyst 5.0.

Table 8SM Top 10 features identified by One-way ANOVA for data obtained in fingerprint analysis of yeast after AMT exposure

Peaks (mz/rt)	*f*-value	*p*-value	-log10(*p*)	FDR
158.88_3.55	209.17	6.149E-8	7.2112	2.6306E-5
344.8_3.55	185.85	9.7973E-8	7.0089	2.6306E-5
135.04_9.49	134.44	3.4946E-7	6.4566	5.2882E-5
140.88_3.55	124.87	4.6641E-7	6.3312	5.2882E-5
112.88_3.55	123.15	4.9238E-7	6.3077	5.2882E-5
474.72_3.54	101.09	1.0629E-6	5.9735	9.489E-5
214.88_3.57	97.215	1.2369E-6	5.9077	9.489E-5
186.88_3.57	92.919	1.474E-6	5.8315	9.8944E-5
549.28_14.27	33.121	7.3565E-5	4.1333	0.0041619
575.36_15.98	27.75	1.4019E-4	3.8533	0.006844

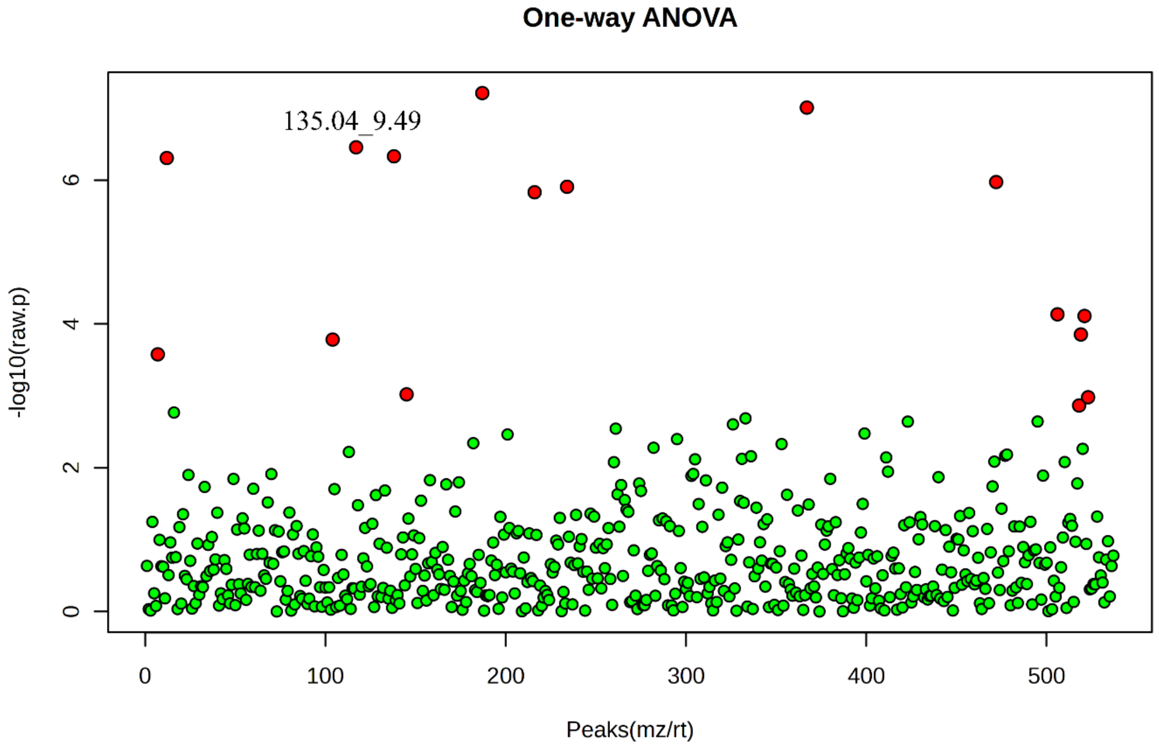

Figure 4SM Important features selected by ANOVA plot with *p*-value threshold 0.05, fingerprint data for AMT exposure.

Table 9SM Top 10 features identified by One-way ANOVA for data obtained in fingerprint analysis of yeast after RMT exposure

Peaks (mz/rt)	*f*-value	*p*-value	-log10(*p*)	FDR
180.08_10.6	668.03	1.61E-18	17.793	2.26E-15
163.12_10.61	139.13	3.99E-13	12.399	2.80E-10
319.28_17.07	90.572	1.13E-11	10.946	5.29E-09
181.12_10.06	85.102	1.83E-11	10.737	6.43E-09
577.44_17.67	79.417	3.13E-11	10.505	8.77E-09
577.44_17.17	62.602	1.94E-10	9.7112	4.54E-08
162.00_4.86	44.054	2.78E-09	8.5557	4.47E-07
308.08_4.83	44.031	2.79E-09	8.554	4.47E-07
348.00_4.32	43.873	2.87E-09	8.5423	4.47E-07
259.04_4.01	41.295	4.51E-09	8.3456	6.33E-07

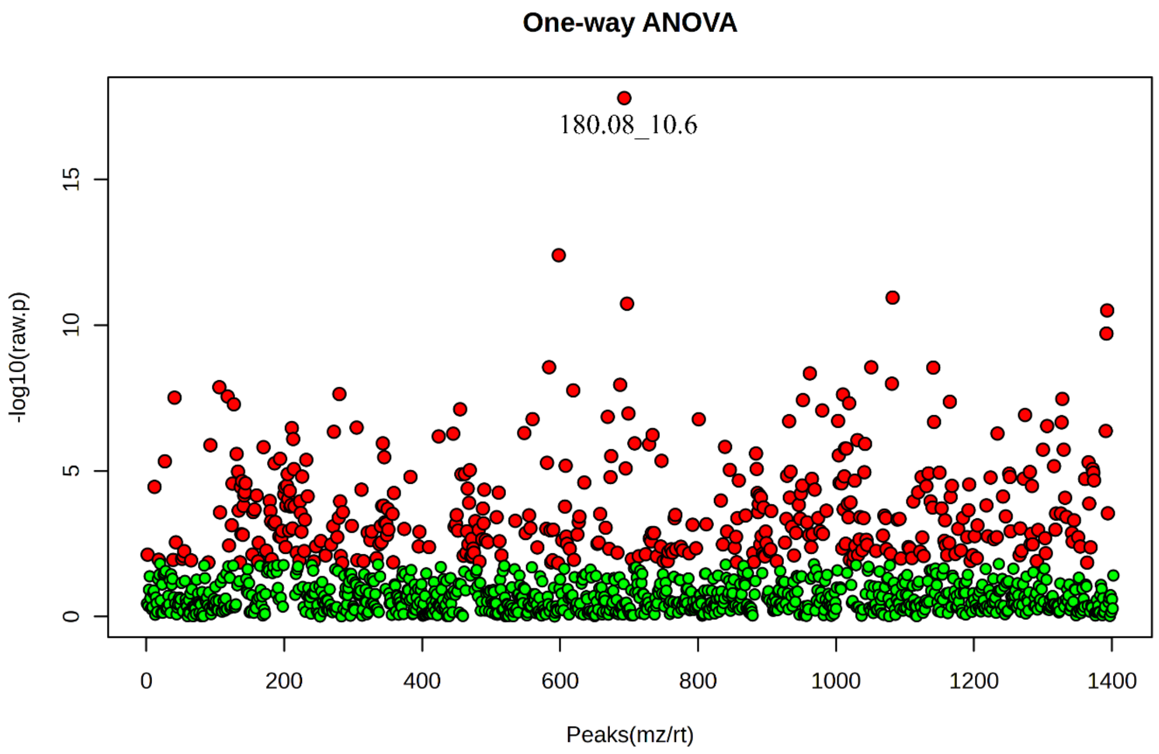

Figure 5SM Important features selected by ANOVA plot with *p*-value threshold 0.05, fingerprint data for RMT exposure.

References

1. Barchanska H., Płonka, J., Nowak, P. & Kostina-Bednarz, M. Metabolic profiles and fingerprints for the investigation of the influence of nitisinone on the metabolism of the yeast *Saccharomyces cerevisiae*. *Sci. Rep*. **13**, 1473 (2023). https://doi.org/10.1038 /s41598-023-28335-3.
